# The Clinical Significance of PIWIL3 and PIWIL4 Expression in Pancreatic Cancer

**DOI:** 10.3390/jcm9051252

**Published:** 2020-04-26

**Authors:** Weiyao Li, Javier Martinez-Useros, Nuria Garcia-Carbonero, Maria J. Fernandez-Aceñero, Alberto Orta, Luis Ortega-Medina, Sandra Garcia-Botella, Elia Perez-Aguirre, Luis Diez-Valladares, Angel Celdran, Jesús García-Foncillas

**Affiliations:** 1 Translational Oncology Division, OncoHealth Institute, Fundacion Jimenez Diaz University Hospital, Av. Reyes Católicos 2, 28040 Madrid, Spain; weiyao.li@quironsalud.es (W.L.); nurigc95@gmail.com (N.G.-C.); alberto.orta@quironsalud.es (A.O.); 2 Pathology Department, University Hospital Gregorio Marañon, C/del Dr. Esquerdo 46, 28007 Madrid, Spain; mgg10167@gmail.com; 3 Pathology Department, Clinico San Carlos University Hospital, C/Profesor Martin Lagos, 28040 Madrid, Spain; luis.ortega@salud.madrid.org; 4 Surgery Department (Pancreatobiliary Unit), Hospital Clínico San Carlos, C/Profesor Martin Lagos, 28040 Madrid, Spain; sandragbotella@hotmail.es (S.G.-B.); eliaperezaguirre@gmail.com (E.P.-A.); lidiez@hotmail.com (L.D.-V.); 5 Hepatobiliary and Pancreatic Surgery Unit, General and Digestive Tract Surgery Department, Fundacion Jimenez Diaz University Hospital, Av. Reyes Católicos 2, 28040 Madrid, Spain; aceldran@idcsalud.es

**Keywords:** PIWI proteins, PIWIL3, PIWIL4, pancreatic cancer, EMT, chemoresistance, motility, HNF4A, survival

## Abstract

P-element-induced wimpy testis (PIWI) proteins have been described in several cancers. PIWIL1 and PIWIL2 have been recently evaluated in pancreatic cancer, and elevated expression of PIWIL2 conferred longer survival to patients. However, PIWIL3’s and PIWIL4’s role in carcinogenesis is rather controversial, and their clinical implication in pancreatic cancer has not yet been investigated. In the present study, we evaluated PIWIL1, PIWIL2, PIWIL3 and PIWIL4 expression in pancreatic cancer-derived cell lines and in one non-tumor cell line as healthy control. Here, we show a differential expression in tumor and non-tumor cell lines of PIWIL3 and PIWIL4. Subsequently, functional experiments with PIWIL3 and/or PIWIL4 knockdown revealed a decrease in the motility ratio of tumor and non-tumor cell lines through downregulation of mesenchymal factors in pro of epithelial factors. We also observed that PIWIL3 and/or PIWIL4 silencing impaired undifferentiated phenotype and enhanced drug toxicity in both tumor- and non-tumor-derived cell lines. Finally, PIWIL3 and PIWIL4 evaluation in human pancreatic cancer samples showed that patients with low levels of PIWIL4 protein expression presented poor prognosis. Therefore, PIWIL3 and PIWIL4 proteins may play crucial roles to keep pancreatic cell homeostasis not only in tumors but also in healthy tissues.

## 1. Introduction

Pancreatic cancer (PC) has arisen as one of the tumors with higher incidence in developed countries. Indeed, the incidence of PC is expected to be higher than breast, prostate or colorectal cancers and to reach the second cause of cancer-related death by 2030 [1]. The 5-year survival rate is 50% when tumors are <2 cm in size and close to 100% for tumors <1 cm [2]; unfortunately, PC is normally asymptomatic, and it is often diagnosed when the tumor has metastasized to distant organs [3]. Adjuvant treatment for complete resected patients (R0) is usually based on Gemcitabine [4], or 5-fluorouracil for six months [5]. Regimens based on Gemcitabine in combination with Nanoalbumin bound-Paclitaxel (Nab-Paclitaxel) is recommended to patients with advanced disease [6]. Nevertheless, PC develops multi-pathways chemoresistance as a result of the interaction between tumor cells, cancer stem cells and the tumor microenvironment [7].

P-element-induced wimpy testis (PIWI) proteins belong to the Argonaute (AGO) family and have been firstly discovered in germline cells [8]. Based on their protein sequence, eight members of the Argonaute family have been classified into two subfamilies: the PIWI subfamily (PIWIL1, PIWIL2, PIWIL3 and PIWIL4) and the AGO subfamily (AGO1, AGO2, AGO3 and AGO4) [9]. The AGO family regulates gene expression through complementary recognition and guidance of short RNAs against their target genes [10]. Recently, it has been reported how PIWI proteins are expressed during the epigenetic remodeling and meiosis of the germline [11]. They also recognize and bind a unique type of non-coding small RNAs called piRNAs (PIWI-interacting RNAs), which constitutes the so-called piRNA-induced silencing complex (piRISC). PIWI proteins have an important role in epigenetic regulation, silencing of transposable elements, protection of genome integrity, gametogenesis and piRNA biogenesis [12]. Indeed, the expression of PIWI proteins promotes some of the hallmarks of cancer such as cell proliferation, genomic integrity, apoptosis, invasion and metastasis [13]. Therefore, an increasing number of studies report their differential expression patterns between healthy and tumor samples and how their modulation affects the behavior of tumor cells. PIWIL1 downregulation drastically reduces the proliferation, invasion and migration of hepatocellular carcinoma cells [14]. Other studies describe how PIWIL1 downregulation in sarcoma inhibits cell growth and allows cell differentiation and support the idea that PIWIL1 tumorigenic activity is due to its ability to regulate DNA hypermethylation [15]. Downregulation of PIWIL1 suppresses cell proliferation, migration and invasion of gastric cancer and lung cancer cells [16,17,18]. These studies sustain the oncogenic features of PIWIL1 and support the idea that PIWIL1 could be used as a target for anticancer therapies. In contrast, other reports showed that overexpression of PIWIL1 decreases proliferation and migration of chronic myeloid leukemia cells through inhibition of expression of matrix metalloproteinase-2 and -9 [19]. Our group has recently described the prognostic role of PIWIL1 and PIWIL2 protein expression in PC, especially PIWIL2 protein, which exhibited higher prognostic potential to predict longer progression-free survival (*p* = 0.029) and longer overall survival (*p* = 0.025). Furthermore, we provided new insight into the link between PIWIL1 and PIWIL2 with the progenitor molecular subtype of PC [20].

PIWIL3 is expressed in stage III epithelial ovarian cancer in both primary tumor and metastatic tissues compared with their adjacent normal tissues (*p* < 0.01), and the expression is higher in the metastatic foci [21]. PIWIL3 is also considered a prognostic biomarker of breast cancer since its upregulation was significantly associated to a short progression-free survival (*p* = 0.01) and a poor overall survival (*p* = 0.02) [22]. Furthermore, PIWIL3 seems to play a crucial role in melanoma progression, and its expression is higher with the higher tumor stage [23]. In gastrointestinal cancers, expression of PIWIL3 was also higher in tumors compared with their paired untransformed tissues [24]. Furthermore, upregulation of PIWIL3 increases proliferation, migration and invasion of gastric cancer cells [24]. In contrast, PIWIL3 seems to play a protective effect due to its overexpression-reduced proliferation, migration and invasion of glioma cells in vitro and decreased tumor size in vivo [25].

The role of PIWIL4 involves chromatin modifications in human somatic cells [26], and it is able to process precursor hairpins to generate several miRNAs in the absence of the endoribonuclease DICER [27]. The lack of PIWIL4 could derive to the development of type 2 diabetes since its downregulation in pancreatic beta cells resulted in defective insulin secretion [28]. However, its function in tumorigenesis is rather controversial. On the one hand, high expression of PIWIL4 is found in tumor tissues of colorectal cancer [29], cervical cancer [30], gastric cancer [31] and primary and metastatic foci of ovarian cancer [21] compared with their adjacent tissues. Its downregulation not only enhanced significantly the apoptotic effect of treatment in Leydig cell tumor [32] but also apoptosis, migration and invasion of breast cancer cells in vitro [33,34]. In hepatocellular carcinoma, the nuclear expression of PIWIL4 together with PIWIL2 has been found to confer worse outcome [35]. On the other hand, other studies have reported that low PIWIL4 expression was significantly associated with a worse prognosis in hepatocellular carcinoma [36], soft tissue sarcoma [37], non-small cell lung cancer [38] and renal cell carcinoma [39]. Low levels of PIWIL4 were also found in hepatocellular carcinoma tissues [36] and in other tumors like breast tumors [22] and non-small cell lung cancer [38] compared to the non-cancerous tissues. Moreover, the lack of PIWIL4 expression caused by CpG island hypermethylation has also been found in testicular tumors [40].

Since PIWIL3 and PIWIL4 expression has not been studied in PC and the functions of PIWI proteins in cancer seem to be rather controversial, we have evaluated the role of PIWIL3 and PIWIL4 expression in pancreatic cells and dissect their prognostic potential in PC.

## 2. Experimental Section

### 2.1. Cell Lines and Cell Culture

The human PC-derived cell lines PANC 04.03(ATCC ref: CRL-2555), PL45(ATCC ref: CRL-2558), BxPC-3(ATCC ref: CRL-1687) and one non-tumor human pancreatic ductal epithelial cell line hTERT-HPNE (ATCC ref: CRL-4023) were purchased and cultured under American Type Culture Collection (ATCC) recommendations. RWP1 and PANC-1 were kindly provided by Dr. Fatima Gebauer (CRG, Barcelona, Spain). RWP1, PANC-1 cells were routinely grown in RPMI supplemented with 10% fetal bovine serum (FBS) and 1% Penicillin-Streptomycin (P/S). All cell lines were maintained at 37 °C in a humidified atmosphere with 5% CO_2_.

### 2.2. Patient Samples

We evaluated the prognostic potential of PIWIL3 and PIWIL4 in a training set and in a validation set of PC samples with tissue microarrays (TMA). TMA of the training set was performed with 44 formalin-fixed, paraffin-embedded tumor samples from BioBank of University Hospital Fundacion Jimenez Diaz—Universidad Autonoma de Madrid (PT13/0010/0012), and the TMA for validation set was constructed with 182 available formalin-fixed and paraffin-embedded tumor samples from BioBank of University Hospital Clinico San Carlos (B.0000725; PT17/0015/0040; ISCIII-FEDER). (Detailed descriptions of all experimental procedures are provided in Supplementary Information 1: Materials and Methods)

## 3. Results

### 3.1. PIWIL3 and PIWIL4 Are Overexpressed in Non-Tumor and Tumor-Derived Cell Lines

**Figure 1:**
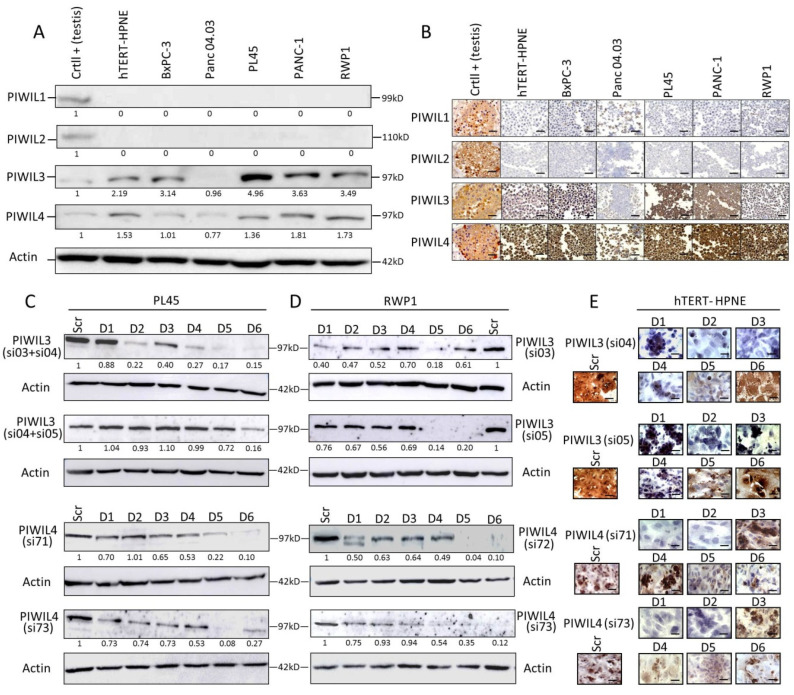
P-element-induced wimpy testis (PIWI) proteins present differential expression in pancreatic cancer (PC), and a late downregulation of PIWIL3 or PIWIL4 in tumor cell lines was found compared to non-tumor cell line. (**A**) Western blot analysis, and (**B**) representative micrographs of immunohistochemical staining of a panel of five human PC-derived cell lines and one non-tumor pancreatic cell line (hTERT-HPNE). A human testis tissue was used as positive control. Two independent downregulations of PIWIL3 (top) and PIWIL4 (bottom) were performed to carry out functional experiments with PL45 (**C**), RWP1 (**D**) and hTERT-HPNE (**E**). Crtl: control. kDa: kilodalton. Scr: Scramble. D1–6: Days 1–6. PIWIL3/Actin or PIWIL4/Actin ratio is represented under each protein band. Scale bar: 50 µm.

All human PIWI proteins were evaluated by Western blot and by immunohistochemistry (IHC) in a panel of five PC-derived cell lines: four from duct-adenocarcinoma differentiation (BxPC-3, Panc04.03, PL45 and RWP1), and one from epithelioid-carcinoma differentiation (PANC-1). Moreover, PIWI proteins were determined in one non-tumor cell line developed from human pancreatic duct transduced with a retroviral expression vector containing the human *TERT* gene (hTERT-HPNE) (Figure 1A,B).

Protein expression was compared with the expression of human testis as positive control. PIWIL1 and PIWIL2 showed very scarce expression in all pancreatic cell lines, not only in the tumor-derived but also in the non-tumor cell lines. PIWIL1 expression in all cell lines was not detected by WB (Figure 1A), although it could be visualized in some cells of BxPc-3 or Panc04.03 by IHC (Figure 1B). Expression levels of PIWIL2 were unnoticeable by both techniques (Figure 1A,B). In contrast, PIWIL3 and PIWIL4 showed overexpression in almost all tumor-derived cell lines, and in the non-tumor pancreatic cell line compared to control (Figure 1A,B). Both PIWIL3 and PIWIL4 exhibited a clear cytoplasmic expression pattern with some nuclear staining (Figure 1B). Panc04.03 was the only PC-derived cell line with the lowest expression levels of PIWIL3 or PIWIL4 (Figure 1A,B). Since PIWIL3 and PIWIL4 are expressed in the immortalized non-tumor pancreatic cell line, we cannot conclude that PIWIL3 or PIWIL4 could act as an oncogene. Then, we wondered whether PIWIL3 or PIWIL4 take part in other mechanisms and which is the response of cells after PIWIL3 or PIWIL4 downregulation in the absence of PIWIL1 and PIWIL2. To this aim, we downregulated PIWIL3 and/or PIWIL4 with two different validated siRNA sequences. The highest expression levels have been shown in two pancreatic ductal adenocarcinoma-derived cell lines (PL45 and RWP1) (Figure 1C,D) and the non-tumor pancreatic cell line (hTERT-HPNE) (Figure 1E). As PL45 showed almost five-fold PIWIL3 expression levels compared with control, and two independent combinations with two different siRNA were necessary to downregulate PIWIL3 (Figure 1C). We also decided to evaluate PIWIL3 or PIWIL4 downregulation on hTERT-HPNE by IHC rather than by Western blot due to the low cellularity that exhibited this cell line. Here, we found that maximum PIWIL3 or PIWIL4 downregulation was achieved later in both tumor cell lines than in non-tumor cell line. Higher PIWIL3 or PIWIL4 downregulation in both tumor cell lines was achieved between the fifth/sixth days (Figure 1C,D) compared with the second day obtained in the non-tumor cell line (Figure 1E).

### 3.2. PIWIL3 and/or PIWIL4 Are Necessary for Cell Motility of Both Non-Tumor and Tumor-Derived Cell Lines

**Figure 2:**
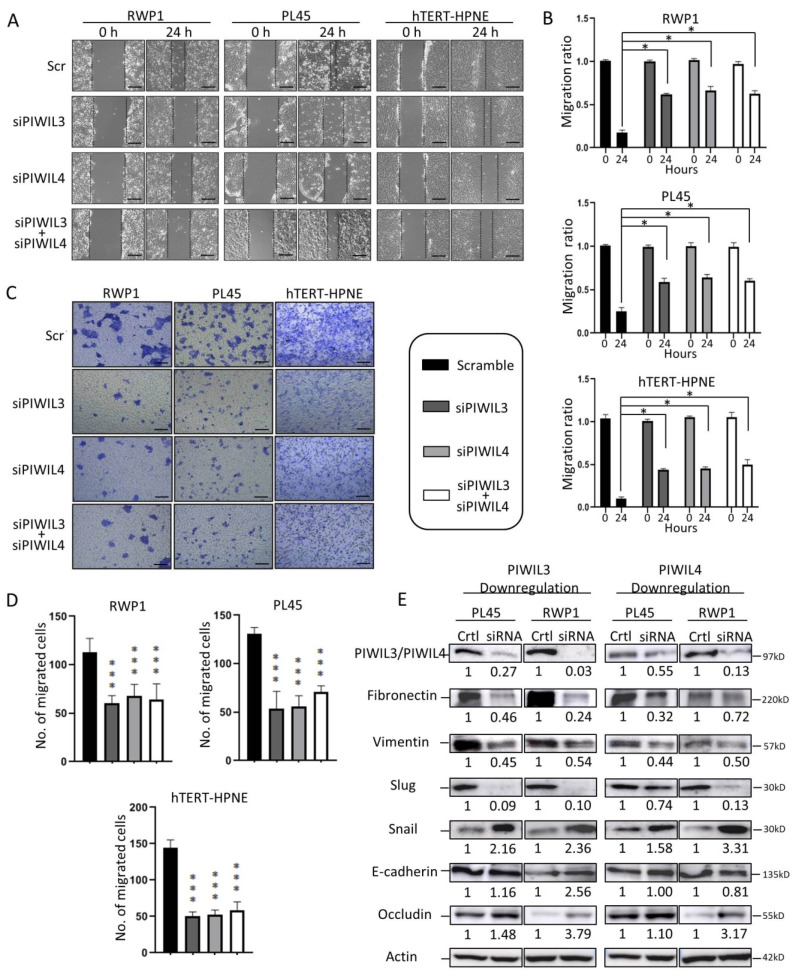
Downregulation of PIWIL3 and/or PIWIL4 decreased motility of PC and non-tumor cell lines through regulation of epithelial-to-mesenchymal transition (EMT). (**A**) Micrographs of wound healing assay showed reduced cell motility after PIWIL3 and/or PIWIL4 silencing in both PC-derived cell lines and in the non-tumor pancreatic-derived cell line. Representative images have been taken at 0 and 24 h after scratching. Broken lines indicate migration heads. (**B**) Statistical analyses of the motility ratio for each cell line according to PIWIL3 and/or PIWIL4 silencing. (**C**) Representative images from Boyden chamber assay of different cell lines taken at 24 h after seeding. (**D**) Statistical analyses of the number of migrated cells for each cell line according to PIWIL3 and/or PIWIL4 silencing. (**E**) Western blot for the expression of PIWIL3 (left) or PIWIL4 (right), Fibronectin, Vimentin, Slug, E-Cadherin and Occludin in PL45 and RWP1 after PIWIL3 or PIWIL4 silencing. The ratio of each protein/Actin ratio is represented under each protein band. Color-coding for each protein downregulation is indicated in the legend box. kDa: kilodalton. Scale bar: 50 µm. *p < 0.05; ***p < 0.001.

Since one of the characteristics of PC is its ability to migrate and metastasize to distant organs, we evaluated the role of PIWIL3 or PIWIL4 in cell motility. Here, we performed functional experiments with two different tumor-derived cell lines and one non-tumor cell line. Interestingly, wound healing assay showed a delay in the motility ratio in all cell lines, normal and tumoral, after PIWIL3 and/or PIWIL4 silencing (Figure 2A).

Statistical analyses compared to control revealed a significant reduction in the motility ratio of all cell lines downregulated for PIWIL3 or PIWIL4 individually or in combination (*p* < 0.05) (Figure 2B). To verify our previous results, a Boyden chamber assay was performed as previously described by Chen [41]. Although all cell lines and scrambles were cultured with the same chemotactic agent (20% FBS), the number of migrating cells decreased significantly after individual PIWIL3 and/or PIWIL4 knockdown alone or in combination compared to scramble (*p* < 0.001) (Figure 2C,D). Interestingly, this fact was not only observed in tumor cell lines but also in the normal cell line, which also decreased its motility after PIWIL3 and/or PIWIL4 downregulation. These results suggest that PIWIL3 and PIWIL4 not only modulate invasiveness of tumor cells but also motility of normal cells, which could impair wound healing processes of adult healthy tissues.

To further study how PIWIL3 and PIWIL4 affect cell motility, we evaluated the expression of different markers involved in epithelial-to-mesenchymal transition (EMT). Interestingly, the mesenchymal proteins Fibronectin and Vimentin reduced their expression after PIWIL3 or PIWIL4 downregulation (Figure 2E). Transition factor Slug highly reduced its protein level after PIWIL3 or PIWIL4 downregulation (Figure 2E). Moreover, epithelial markers like Occludin increased its expression after PIWIL3 or PIWIL4 knockdown in both cell lines, while E-Cadherin raised its protein levels only after PIWIL3 silencing (Figure 2E). These results highlight the role of PIWIL3/PIWIL4 in cell motility and wound healing of pancreatic cells through regulation of EMT factors. Taking into consideration that downregulation of PIWIL3 or PIWIL4 reverses EMT of normal cell line, the modulation of these two proteins could affect adult tissue reconstruction after trauma, toxic treatments or inflammatory processes.

### 3.3. Downregulation of PIWIL3 and/or PIWIL4 Impairs Undifferentiated Phenotype

**Figure 3:**
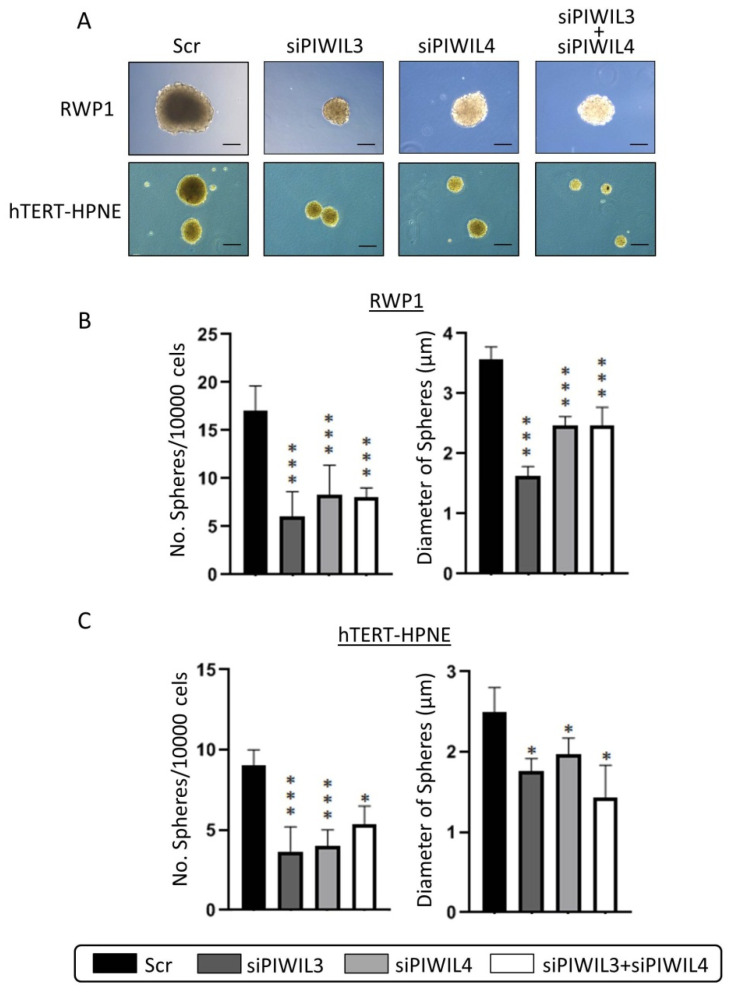
PIWIL3 and PIWIL4 impair undifferentiated phenotype. (**A**) Representative micrographs of undifferentiated pancreatic spheres derived from RWP1 and hTERT-HPNE transfected with siRNA for PIWIL3 (siPIWIL3) or PIWIL4 (siPIWIL4) downregulation individually or in combination. (**B**) Statistical analyses of number and diameter of spheres according to PIWIL3 and/or PIWIL4 downregulation of RWP1 cell line. (**C**) Statistical analyses of number and diameter of spheres according to PIWIL3 and/or PIWIL4 downregulation of the non-tumor hTERT-HPNE cell line. Color-coding for each protein downregulation is indicated in the legend box. Scr: scramble. Scale bar: 50 µm. * *p* < 0.05; *** *p* < 0.001.

Following with functional experiments with PIWIL3 and/or PIWIL4 downregulation, we evaluated the ability of both tumor and non-tumor pancreatic derived cell lines to form pancreatic spheres in stem cell enrichment culture media (Figure 3A).

PL45 was not able to dedifferentiate, and to the best of our knowledge, no detailed research reached PL45 dedifferentiation. The spheres observed from scramble controls ranged from 2 to 4 μm of diameter and formed between 10 and 20 spheres per 10,000 seeded cells. Non-tumor cell line presented the lowest number of spheres and the lowest sphere diameter in control conditions. Remarkably, we observed that PIWIL3 and/or PIWIL4 knockdown dropped drastically the number and diameter of spheres of tumor cell line RWP1 (*p* < 0.001) (Figure 3B). However, the same effect was observed on the non-tumor cell line, hTERT-HPNE, not only in the number of spheres (*p* < 0.001) but also in their diameter (*p* < 0.05) (Figure 3C). These results suggest the role of PIWIL3 and PIWIL4 in the maintenance of undifferentiated phenotype of pancreatic cells; however, it seems not to be only necessary for neoplastic cells, but also for normal cells differentiation. These results hamper the clinical use of PIWIL3 or PIWIL4 modulation in PC patients because it may disrupt the dedifferentiation mechanism not only of tumor cells but also of other healthy tissues and could lead to a severe medical condition for patients.

### 3.4. PIWIL3 and PIWIL4 Downregulation Potentiate the Cytotoxic Effect of Chemotherapies

**Figure 4:**
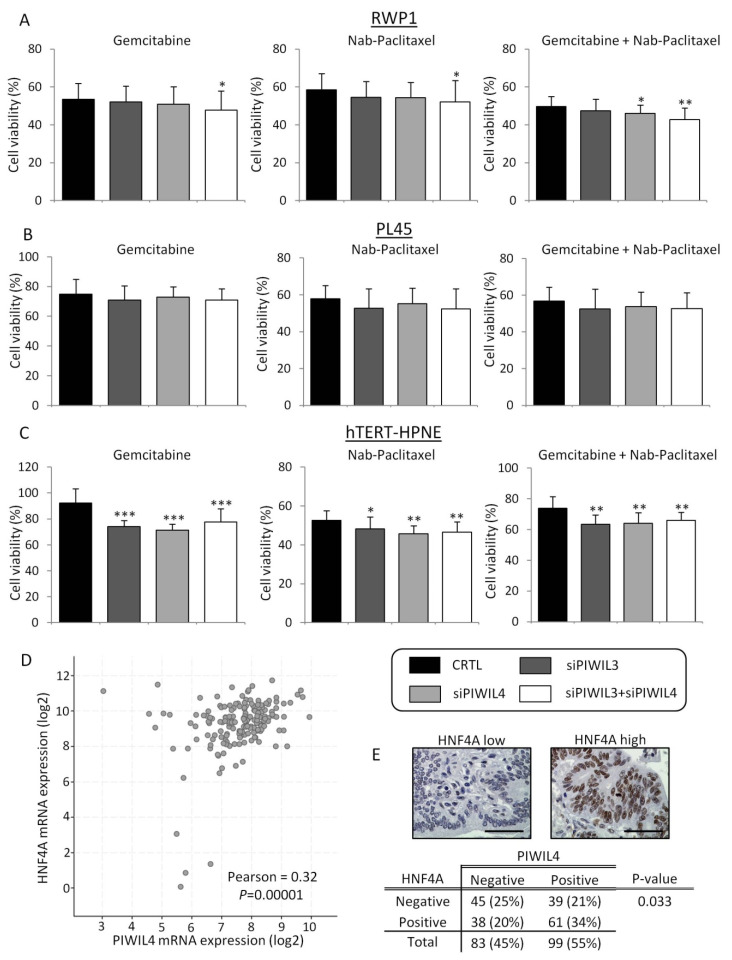
PIWIL3 and PIWIL4 downregulation potentiates the cytotoxic effect of chemotherapy. (**A**) Cell viability analyses after PIWIL3 and/or PIWIL4 silencing according to Gemcitabine (left) or Nab-Paclitaxel (center) individual treatments or in combination (right) of RWP1 cell line, PL45 (**B**) and hTERT-HPNE (**C**) cell lines. (**D**) Scatterplot and statistical analysis of HNF4A mRNA expression (y axis) and PIWIL4 mRNA expression (x axis) of 178 patient cohort from The Cancer Genome Atlas (TCGA). (**E**) Representative micrographs of HNF4A low expression (top-left) and high expression top-right). Statistical association between HNF4A and PIWIL4 protein expression of 182 PC samples (bottom). Color-coding for each protein downregulation is indicated in the legend box. Scale bars: 50 µm. * *p* < 0.05; ** *p* < 0.01; *** *p* < 0.001.

Gemcitabine is one of the gold standard adjuvant treatments for PC management, alone or in combination with Nab-Paclitaxel. Therefore, we wondered whether PIWIL3 and/or PIWIL4 regulate response to these chemotherapies. To evaluate the cytotoxicity of these two factors, tumor and normal cell lines were treated with Gemcitabine or Nab-Paclitaxel individually or in combination after PIWIL3 and/or PIWIL4 knockdown. Then, logarithmically growing tumor-derived cell lines, RWP1 and PL45, and normal cell line, hTERT-HPNE, were treated with previously determined IC_50_ doses of Gemcitabine or Nab-Paclitaxel (Supplementary file). To determine doses for treatment combination for each cell line, IC_25_ dose of Nab-Paclitaxel was set due to its high toxicity, and different concentrations of Gemcitabine were tested to achieve 50% of cell death as previously reported by Awasthi N. et al. [42]. Individual protein downregulation was not enough to achieve an effect, and PIWIL3 and PIWIL4 double downregulation were necessary to decrease significantly cell viability of RWP1 after single treatments (*p* = 0.023 for Gemcitabine; *p* = 0.038 for Nab-Paclitaxel). PIWIL4 downregulation per se achieved a significant effect on the combined treatment (*p* = 0.038); although, double downregulation achieved the maximum effect (*p* = 0.001) (Figure 4A). In contrast, neither PIWIL3 nor PIWIL4 knockdown affected cytotoxicity of PL45 cell line, neither with individual treatments nor in combination (Figure 4B).

On the other hand, non-tumor cell line hTERT-HPNE initially presented a complete resistance to Gemcitabine; then, functional experiments were performed with the highest concentration of Gemcitabine tested (250 µM). This concentration was 42,000 times higher than IC_50_ concentration of Gemcitabine for RWP1 and 700 times higher than IC_50_ concentration of Gemcitabine for PL45. Furthermore, IC_50_ dose of Nab-Paclitaxel for non-tumor cell line (235 µM), which was 21 times higher than IC_50_ dose of Nab-Paclitaxel for RWP1 and 1.6 times higher than for PL45. Interestingly, the highest effect of all treatments was observed in the non-tumor derived cell line. Indeed, PIWIL3 and/or PIWIL4 silencing overcame Gemcitabine resistance of the non-tumor cell line (*p* < 0.001), and significantly increased the other treatment effects (*p* = 0.003 for Nab-Paclitaxel; *p* = 0.001 for Gemcitabine + Nab-Paclitaxel) (Figure 4C). Therefore, these results support PIWIL3 and PIWIL4 as crucial factors in chemoresistance of PC tumor cells and in the toxicity of normal cells. However, from a clinical point of view, depletion of PIWIL3 or PIWIL4 proteins with target therapies should be done with great care due to the potential high toxicity and adverse events that they could bring to PC patients.

In order to dissect one of the underlying mechanisms whereby PIWIL3 or PIWIL4 expression confers chemoresistance, we evaluated the link between these two proteins and hENT1, which is responsible for Gemcitabine uptake and effect on cells [43]. For this, we used 178 available expression profile data from a 186-patient dataset from The Cancer Genome Atlas (TCGA, Firehose Legacy), and statistical correlation was assessed using cBioPortal [44,45]. In this first attempt, piwil3 or piwil4 showed no correlation with hEnt1 at mRNA level (*p* = 0.26 and *p* = 0.19, respectively). Another factor that drives cytotoxicity of tumor cells is HNF4A. It has been previously described to be a negative regulator of hENT1 and necessary for cell proliferation and drug resistance in PC [46]. Then, we assessed the correlation between piwil3 or piwil4 and hnf4a; however, piwil3 mRNA expression did not show any connection with hnf4a at the mRNA level (*p* = 0.36). Interestingly, mRNA analysis showed a moderate positive correlation between piwil4 and hnf4a (r = 0.32; *p* = 0.00001) (Figure 4D). To validate this result, we stained by IHC 182 PC patient samples with anti-HNF4A antibody. HNF4A exhibited a clear nuclear staining and a marked differential expression pattern between samples (Figure 4E, top). The statistical analysis revealed a link between PIWIL4 and HNF4A at the protein level in patient samples (*p* = 0.033)(Figure 4E, bottom). We also assessed an association between PIWIL3 and HNF4A at the protein level. Although no association was found, statistical analysis revealed a high trend towards significance (*p* = 0.080). These results highlight a connection between PIWIL3 and PIWIL4 with HNF4A factor, which could explain a feasible mechanism of chemoresistance of PC cells and cytotoxicity of normal cells.

### 3.5. Low Expression of PIWIL4 Is a Poor Prognosis Factor of Pancreatic Cancer Patients

To study the prognostic potential of PIWIL3 or PIWIL4 in PC, we evaluated their protein expression levels in a cohort composed of 44 patients from Fundacion Jimenez Diaz Hospital. To assess the survival analysis all samples with positive margins of resection (R1) were excluded from the study (*n* = 7 patients) (Table 1).

**Figure 5:**
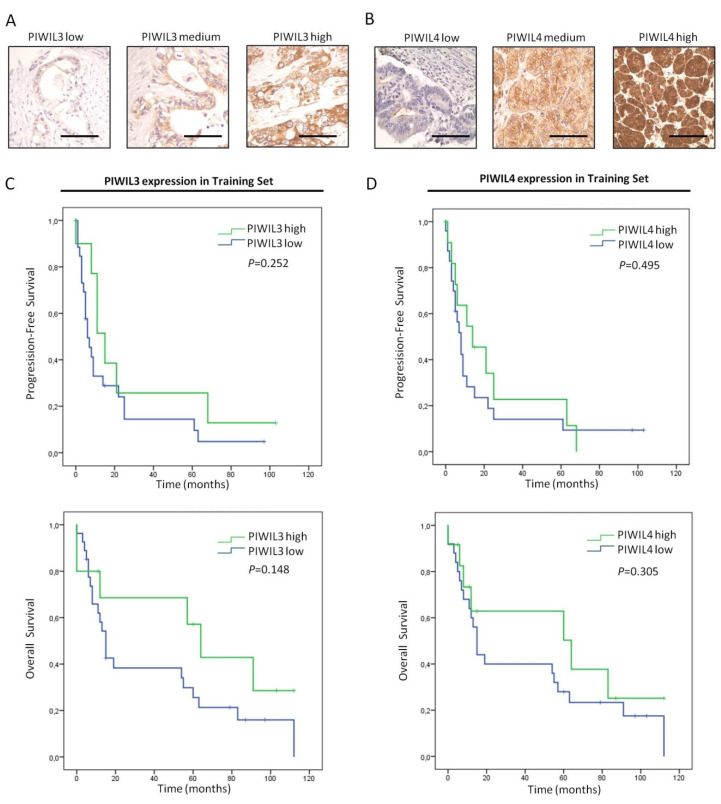
Prognostic impact of PIWIL3 or PIWIL4 in PC patients from the training set. (**A**) Representative micrographs of tumors with low (left), intermediate (middle) and high PIWIL3 expression (right). (**B**) Representative micrographs of tumors with low (left), intermediate (middle) and high PIWIL4 expression levels (right). (**C**) Kaplan–Meier curves according to PIWIL3 protein expression for both progression-free (top) and overall survival (bottom). (**D**) Kaplan–Meier curves according to PIWIL4 protein expression for both progression-free (top) and overall survival (bottom). *p*-values were obtained by log-rank test. Scale bars: 50 µm.

Immunohistochemical staining of patient samples showed differential expression levels of PIWIL3 and PIWIL4. All the samples that stained positively for PIWIL3 exhibited a cytoplasmic localization, especially in those cases with high PIWIL3 expression (Figure 5A). The expression pattern of PIWIL4 was limited to cytoplasm and cell membrane of tumor cells, and no positive nuclear staining was found (Figure 5B). Survival analyses were assessed with this data set. Nevertheless, neither PIWIL3 nor PIWIL4 associated significantly with progression-free or overall survival of PC patients (Figure 5C,D). However, although statistical analyses revealed no significant association between these PIWI proteins and prognosis, we found that patients with low expression levels of PIWIL3 or PIWIL4 presented shorter progression-free and overall survival than high levels of both proteins. The mean progression-free survival of patients with low PIWIL3 expression was 17 months (95% CI = 7–27 months), while the mean time-to-progression of high PIWIL3 expression was 30 months (95% CI = 6–54 months) (Figure 5C, top). Concerning overall survival, patients with low PIWIL3 expression exhibited a mean survival of 37 months (95% CI = 22–53 months), and those with high PIWIL3 expression lived a mean of 62 months (95% CI = 33–90 months) (Figure 5C, bottom). Similarly, low PIWIL4 expression presented shorter mean progression-free and overall survival than high-expression patients. The mean progression-free survival of patients with low PIWIL4 expression was 19 months (95% CI = 6–31 months), while the mean time-to-progression of high PIWIL4 expression was 23 months (95% CI = 8–39 months) (Figure 5D, top). Furthermore, patients with low PIWIL4 expression presented shorter overall survival (mean = 39 months; 95% CI = 23–56 months) than patients with high PIWIL4 expression (mean = 56 months; 95% CI = 30–82 months) (Figure 5D, bottom).

One of the possible reasons that may justify the lack of statistical significance of these analyses could be the limited sample size of the study. Therefore, we evaluated the expression of PIWIL3 and PIWIL4 in a larger cohort composed of 182 patients samples from Clinico San Carlos Hospital. As before, all samples with positive margins of resection were excluded from the study (*n* = 54 patients) (Table 2).

**Figure 6:**
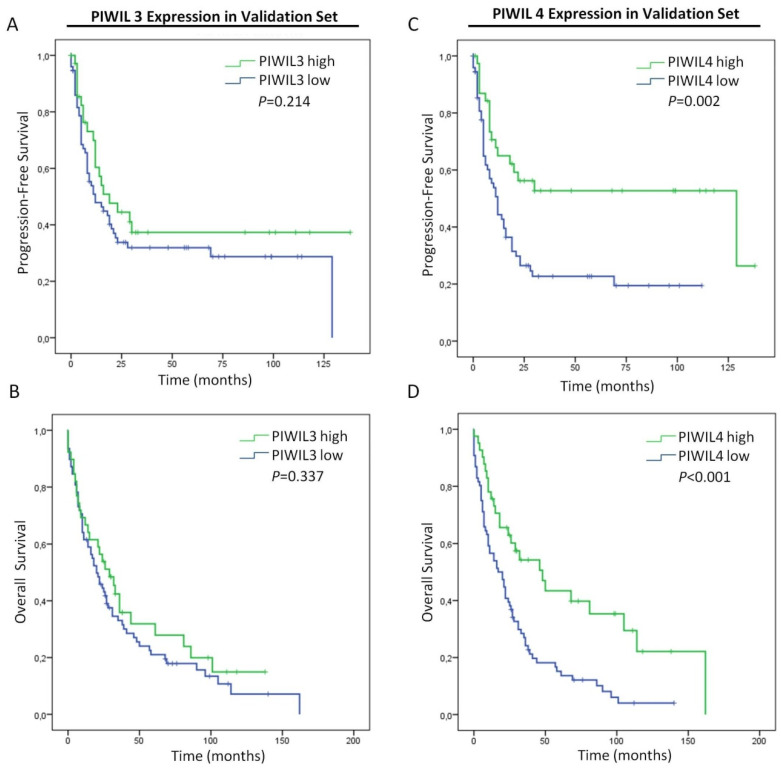
Prognostic impact of PIWIL3 or PIWIL4 in PC patients from the validation set. (**A**) Kaplan–Meier curves according to PIWIL3 protein expression for progression-free survival. (**B**) Kaplan–Meier curves according to PIWIL3 protein expression for overall survival. (**C**) Kaplan–Meier curves according to PIWIL4 protein expression for progression-free survival. (**D**) Kaplan–Meier curves according to PIWIL4 protein expression for overall survival. *p*-values were obtained by log-rank test.

We assessed survival analyses with patients with available data of progression-free survival (*n* = 113) or overall survival (*n* = 118). Here, PIWIL3 expression did not associate either with progression-free survival (*p* = 0.214) or overall survival (*p* = 0.337) (Figure 6A,B). Thus, these results led us to exclude PIWIL3 expression as a prognostic biomarker for PC. Interestingly, those PC patients with low expression of PIWIL4 presented not only a shorter progression-free survival (*p* = 0.002) but also a shorter overall survival (*p* < 0.001) than patients with high expression levels (Figure 6C,D). Here, patients with low PIWIL4 expression showed a mean progression-free survival of 31 months (95% CI = 20–41 months), while patients with high PIWIL4 expression presented a mean progression-free survival of 75 months (95% CI = 54–96 months) (Figure 6C). Overall survival of patients with low PIWIL4 expression presented a mean of 29 months (95% CI = 21–37 months), while that of patients with high PIWIL4 expression was significantly longer with a mean of 68 months (95% CI = 46–89 months) (Figure 6D).

In order to validate the prognosis potential of PIWIL4 expression with respect to other clinico-pathological characteristics, we performed a Cox proportional hazards model for both progression-free and overall survival of patients (Table 3). The univariate analysis for progression-free survival revealed that patients with a low expression of PIWIL4 showed a higher risk of recurrence after surgery compared with patients with high expression (hazard ratio (HR) = 1.979; 95% CI: 1.178–3.325; *p* = 0.010). As survival curves confirmed previously, PIWIL3 did not raise significance to predict progression-free survival (*p* = 0.227). Other pathological characteristics that associated significantly with high risk of progression in the univariate analysis were tumor size (HR = 3.023; 95% CI: 1.413–6.465; *p* = 0.004), T stage (HR = 1.682; 95% CI: 1.033–2.738; *p* = 0.037), tumor stage (HR = 1.866; 95% CI: 1.105–3.151; *p* = 0.020) and neural invasion (HR = 1.757; 95% CI: 1.027–3.007; *p* = 0.040). In the multivariate analysis, low PIWIL4 expression remained statistically significant for a higher risk of progression (HR = 2.036; 95% CI: 1.025–4.044; *p* = 0.042) together with tumor size (HR = 3.095; 95% CI: 1.237–7.744; *p* = 0.016). Univariate analyses for overall survival also revealed low expression of PIWIL4 as a high risk factor (HR = 2.093; 95% CI: 1.344–3.260; *p* = 0.001). Other clinico-pathologic characteristics that associated significantly with shorter overall survival were T stage (HR = 1.679; 95% CI: 1.110–2.540; *p* = 0.014), tumor stage (HR = 1.795; 95% CI: 1.148–2.807; *p* = 0.010), lymph nodes positive (HR = 1.573; 95% CI: 1.025–2.414; *p* = 0.038) and neural invasion (HR = 1.658; 95% CI: 1.060–2.593; *p* = 0.027). However, the only clinical variable that associated significantly with reduced overall survival in the multivariate analysis was low PIWIL4 expression (HR = 2.185; 95% CI: 1.313–3.636; *p* = 0.003) (Table S3). Thus, these results highlight the detrimental role of low expression of PIWIL4 and allow the identification of two different risk subgroups of PC patients to be managed with differential treatment strategies to improve survival.

In view of these results, we verified whether PIWIL3 or PIWIL4 could be related to any of the pathological characteristics registered in our study (Table S4). In this analysis, low levels of PIWIL3 associated significantly with neural invasion (*p* = 0.050). Low PIWIL4 expression associated significantly with female patients (*p* = 0.050). Furthermore, a higher percentage of patients with T3 tumors associated significantly with low PIWIL4 expression (*p* = 0.020); the same occurred with neural invasion and low PIWIL4 expression (*p* = 0.019) (Table 4). These results suggest the lack of PIWIL4 expression as a deleterious effect in PC and support previous survival results.

## 4. Discussion

PC is an extremely lethal malignancy, in which an early diagnosis is crucial to increase patient survival. Therefore, molecular biomarkers will play an important role in the future management of this neoplasm. To date, the only biomarkers approved by the Food and Drug Administration (FDA) for PC are preoperative levels of CA19-9; however, the applicability of this biomarker has been questioned due to the fact that the biliary obstruction can also increase CA19-9 levels, not to mention that up to 10% of the population cannot synthesize this antigen [47]. Therefore, new biomarkers that combine high sensitivity and specificity are needed in the clinical management of PC. Recently, novel proteins called PIWI have been discovered, and their expression was found in several types of tumors; thus, these factors may provide new perspectives in the clinical practice of PC [12]. In the present study, we have evaluated the expression of the four members of the PIWI family in PC-derived cell lines and one normal pancreatic cell line used as control. Interestingly, both PIWIL1 and PIWIL2 presented nearly undetectable expression levels in all cell lines. Indeed, this fact could be explained by the presence of CpG islands in the promoter region of *PIWIL1* [48] and *PIWIL2* [49]. It has been reported that downregulation of PIWIL1 and PIWIL2 by promoter CpG island hypermethylation has been observed in other types of tumors like testicular or non-small cell lung cancer [38]. It has also been described how PIWIL1 downregulation regulates migration of Schwann cells for peripheral nerve regeneration after injury [50]. Since we found low levels of PIWIL1 and PIWIL2 in a pancreatic normal cell line, this event seems not to be exclusive of tumor cells. In fact, these genes play crucial roles in spermatogenesis, and their downregulation impairs germ cell development that might associate with male infertility [51].

On the other hand, PIWIL3 and PIWIL4 showed higher protein levels and a differential expression pattern throughout cell lines, which includes a non-tumor cell line. This first attempt implied that PIWIL3 and PIWIL4 might not act as an oncogene in PC. Nevertheless, the role of PIWIL3 and PIWIL4 in tumorigenesis is rather controversial. For this, we decided to evaluate their role with functional experiments in tumor cell lines and a non-tumor cell line as normal control. Some studies have reported the expression of these proteins with oncogenic features; e.g., one study described how cancer cells re-express PIWIL3 to promote cancer cell growth [52]. Other research highlighted that PIWIL3 and PIWIL4 presented oncogenic potential in several types of cancers [13]. In contrast, PIWIL3 exhibited a protective effect in glioma cells [25], and low expression of PIWIL4 has been found in tumor cells from hepatocellular carcinoma [36], breast cancer [22] and non-small cell lung cancer [38]. Therefore, the role of PIWIL3 and PIWIL4 in tumor initiation and development remains still unclear. In our functional experiments, we were able to evaluate cell response to PIWIL3 and/or PIWIL4 downregulation. Moreover, the inclusion of a non-tumor cell line in these experiments led us to discern between a true oncogenic role and a normal cell function. Our experiments, designed to evaluate cell motility, chemoresistance and undifferentiated phenotype, revealed that the effect observed after PIWIL3 and/or PIWIL4 downregulation in tumor cells were also shown by the non-tumor cell line. Here, we observed how PIWIL3 and PIWIL4 knockdown decreased motility of both tumor and normal cells through a mesenchymal arrest in favor of the epithelial phenotype. This reduction of the cell motility by PIWIL4 downregulation has previously been described in breast cancer cells through an impairment of Vimentin and N-Cadherin [33]. However, this study only provided evidence of migration delay in MCF-7 tumor cell line but not in a non-tumor cell line. Then, it is still unknown whether PIWIL4 downregulation exclusively affects cell motility of breast cancer cells or also impairs motility of normal cells. For this concern, it has been reported how PIWIL2 regulates invasion abilities of prostate cancer cells through modulation of EMT protein expression [53]. HPV16 is also able to increase PIWIL2 levels to increase proliferation and invasion of cervical cancer cells [54]. However, not only do PIWI proteins play a role as invasion promoting factors, but also their associated piRNAs. It has been described how downregulation of piRNA-36712 promotes invasion and migration of tumor cells; thus, it is considered a potential tumor suppressor in breast cancer [55]. Another study supports the tumor-suppressive properties of piR-823 because its upregulation inhibits tumor cell growth in gastric cancer models [56]. In addition, piR-823 downregulation suppressed cell proliferation of colorectal cancer cells by a direct modulation of the transcriptional activity of HSF1 [57]. Other functional experiments have demonstrated that piR-651 promotes tumor formation in non-small cell lung cancer mediated by Cyclin D1 and CDK4 [58]. To the best of our knowledge, we have described for the first time the implication of PIWIL3 and PIWIL4 in cell motility through EMT modulation of tumor and non-tumor pancreatic cells. From a clinical point of view, this connection between PIWIL3/PIWIL4 and EMT should be managed carefully since EMT is the most critical mechanism by which adult tissues, including pancreatic β-cells, are repaired after inflammatory, toxic or trauma injuries [59,60,61].

Many works have reported that PIWI proteins have the ability to regulate transposable elements to maintain genomic stability of stem cells [62]. In our functional studies, we observed a diminished undifferentiated phenotype of pancreatic cells, and we found a decrease in the number and size of pancreatic stem-cell-like spheres after PIWIL3 and/or PIWIL4 downregulation. This result supports the role of PIWIL3/PIWIL4 in the maintenance of undifferentiated phenotype both in tumor and in normal cells, as was previously observed in normal spermatogenesis of mammals [63]. Moreover, downregulation of PIWIL2 decreased proliferation and survival of breast cancer stem cells through a decrease in the protein levels of STAT3, BCL-XL and Cyclin D1 [64]. This link between PIWI proteins and undifferentiated phenotype has also been demonstrated when downregulation of PIWI proteins impaired whole-body regeneration of certain marine organisms [65]. Hence, the role of PIWIL3/PIWIL4 seems not to be exclusive of tumorigenesis and suggests a crucial function in fundamental tissue maintenance.

Since expression of PIWI proteins increased resistance to drugs in cervical cancer [66] and in non-small cell lung cancer [67], we decided to evaluate whether PIWIL3 or PIWIL4 were able to modulate chemoresistance of PC. Here, we described how PIWIL3 and PIWIL4 downregulation increases the effect of the gold standard chemotherapies against PC. Surprisingly, PL45 cell line showed no effect after individual or combined downregulation. However, the lack of effect in PL45 could be explained not only by its mutations in *KRAS*, *TP53* or *DPC4*, which are commonly found in PC, but also by its mutation in *BRCA2* gene, which could confer chemoresistance in PC as recently described by Wang et al. [68]. As we observed and as previously reported in the literature, non-tumor cell line hTERT-HPNE showed Gemcitabine resistance [69]. Nevertheless, it reverted completely its chemoresistance after PIWIL3 and/or PIWIL4 knockdown and significantly increased the effect of Gemcitabine alone or in combination with Nab-Paclitaxel. However, this statistically significant drug response exhibited after double downregulation achieved neither additive nor synergic effect compared with individual protein downregulation in the presence of single treatment or combination. The fact that PIWIL3 and/or PIWIL4 downregulation increased considerably drug response on the normal cell line does not make modulation of PIWIL3 or PIWIL4 suitable for future drug design against PC. This effect on normal cells could imply higher toxicity and adverse events, which could compromise tolerability and safety of patients. In order to explain the link between these two PIWI proteins and chemoresistance, we explored factors related to Gemcitabine or Nab-Paclitaxel resistance in PC. Hepatocyte Nuclear Factor Alpha (HNF4A) appeared rapidly as a potential factor that may account for this finding. HNF4A is overexpressed in hepatocytes, enterocytes and pancreatic β-cells. It also ensures expression of intermediary genes required for metabolism of glucose and lipids, and it is necessary for cell differentiation [70]. In PC, high expression levels of HNF4A have been correlated with poor prognosis. HNF4A has been described as conferring chemoresistance in other types of tumors like breast cancer, where it has been the most upregulated gene after hypoxic conditions and led to a higher Doxorubicin resistance [71]. Indeed, a synthetic HNF4A antagonist is under investigation to selectively eradicate cancer cells [72]. Moreover, the mechanism of HNF4A to confer chemoresistance to Gemcitabine is through (a) direct regulation of hENT1, which is responsible for Gemcitabine uptake of tumor cells [46]. At first glance, neither PIWIL3 nor PIWIL4 exhibited a correlation with hENT1. Nevertheless, a high trend towards significance was found between PIWIL3 and HNF4A at the protein level, and a statistically significant correlation was found between PIWIL4 and HNF4A both at mRNA and at the protein level. Therefore, these results support the role of these PIWI proteins as crucial factors for regulation of chemotherapy uptake of cells.

Finally, we assessed survival analyses by staining PIWIL3 or PIWIL4 in PC samples. We were struck in particular by the fact that PIWIL3 and PIWIL4 were expressed in pancreatic normal tissues [73,74]; consequently, our hypothesis as oncogenes was found baseless and was simply discarded. Furthermore, survival analyses revealed that low expression of PIWIL4 associated significantly with both shorter progression-free and overall survival. These results suggested a deleterious effect of low levels of PIWIL4. Since PC is a deadly disease and survival of patients is rather limited, our findings allow the identification of two different risk subgroups of PC patients that can be clinically managed independently to improve survival. Only tumor size higher than 2 cm emerged as statistically significant together with low PIWIL4 expression in Cox multivariate analysis for progression-free survival. This result could be expected, since tumor size at diagnostic is closely related to survival. It has been reported that the 5-year survival rate is around 50% when tumors are below 2 cm [75] and close to 100% when tumors are below 1 cm [76]. Moreover, we found that a higher percentage of patients with low PIWIL4 expression exhibited a link with T3 tumors and neural invasion compared with those with high PIWIL4 expression.

On the other hand, the fact that low levels of PIWIL4 are related to reduced cell motility seemed to go against our results that suggest it as a poor prognostic biomarker of PC. However, our results suggest that the lack of PIWIL4 could increase treatment toxicity and adverse events to patients, an impaired tissue repair driven by a delay in cell motility through EMT reversion, and a default on cell differentiation. All these mechanisms could retard the healing process of PC patients and lead to shorter progression-free and overall survival.

## 5. Conclusions

In our study, we have compiled some functional experiments and survival analysis according to PIWIL3 or PIWIL4 expression to dissect the role of these proteins in PC. Our findings support PIWIL3 and PIWIL4 as crucial factors in the regulation of cell motility, stem cell maintenance and drug resistance both in tumor and healthy pancreatic cells. Moreover, low PIWIL4 expression is able to predict shorter survival of PC patients. These results provide new insights into the knowledge of PIWI proteins functions and their controversial role in tumorigenesis.

## Figures and Tables

**Table 1:** Clinico-pathologic characteristics of completed resected R0 pancreatic cancer patients from the training set.

Clinical Characteristics	*N*	%	Clinical Characteristics	*N*	%
Age			Neural invasion		
<65 years	16	43	No	12	32
>65 years	21	57	Yes	25	68
Gender			Lymph nodes involved		
Male	21	57	N0	14	38
Female	16	43	N1	23	62
Size			Adjuvant treatment		
<2 cm	20	54	No	21	57
>2 cm	17	46	Yes	14	38
Stage			N/A	2	5
I	9	24	pT		
II	28	76	T1	6	16
Grade			T2	5	14
High	30	81	T3	26	70
Low	7	19	N/A	3	2
Vascular invasion					
No	12	32	Total	37	100
Yes	25	68			

N: number of patients; N/A: not available; cm: centimeters.

**Table 2:** Clinico-pathologic characteristics of complete resected R0 pancreatic cancer patients from the validation set.

Clinical Characteristics	*N*	%	Clinical Characteristics	*N*	%
Age			Grade		
<65 years	25	20	High	19	15
>65 years	103	80	Low	105	82
Gender			N/A	4	3
Male	63	49	Vascular invasion		
Female	65	51	No	75	59
Diabetes Mellitus			Yes	43	33
No	88	69	N/A	10	8
Yes	33	26	Neural invasion		
N/A	7	5	No	47	37
Adjuvant treatment			Yes	71	55
No	75	58	N/A	10	8
Yes	24	19	pT		
N/A	29	23	T1	30	23
Size			T2	44	35
<2 cm	31	24	T3	51	40
>2 cm	69	54	N/A	3	2
N/A	28	22	Lymph nodes involved		
Stage			N0	70	55
I	46	36	N1	51	40
II	74	58	N/A	7	5
N/A	8	6	Total	128	100

N: number of patients; N/A: not available; cm: centimeters.

**Table 3:** Uni- and multivariate proportional hazards model for progression-free and overall survival of patients from the validation cohort.

	Univariate PFS (95% CI)				Univariate OS (95% CI)			
	HR	Lower	Upper	*p*	HR	Lower	Upper	*p*
Age (< 65 years vs. > 65 years)	1.060	0.604	1.860	0.840	1.198	0.723	1.986	0.484
Gender (Male vs. Female)	1.494	0.920	2.425	0.104	1.182	0.785	1.778	0.423
Diabetes Mellitus (No vs. Yes)	1.070	0.614	1.864	0.811	1.113	0.694	1.784	0.658
Adjuvant treatment (Yes vs. No)	1.016	0.556	1.857	0.959	1.226	0.781	2.094	0.456
Size (<2 cm vs. >2 cm)	3.023	1.413	6.465	0.004	1.255	0.754	2.087	0.382
pT (I / II vs. III)	1.682	1.033	2.738	0.037	1.679	1.110	2.540	0.014
Stage (I vs. II)	1.866	1.105	3.151	0.020	1.795	1.148	2.807	0.010
Grade (low vs. high)	1.406	0.695	2.845	0.343	1.221	0.664	2.245	0.522
Lymph nodes involved (No vs. Yes)	1.548	0.943	2.540	0.084	1.573	1.025	2.414	0.038
Vascular invasion (No vs. Yes)	1.348	0.807	2.252	0.254	1.481	0.959	2.287	0.077
Neural invasion (No vs. Yes)	1.757	1.027	3.007	0.040	1.658	1.060	2.593	0.027
PIWIL3 (high vs. low)	1.380	0.819	2.327	0.227	1.237	0.798	1.917	0.342
PIWIL4 (high vs. low)	1.979	1.178	3.325	0.010	2.093	1.344	3.260	0.001
	**Multivariate PFS (95% CI)**				**Multivariate OS (95% CI)**			
Size (<2 cm vs. > 2 cm)	3.095	1.237	7.744	0.016				
pT (I / II vs. III)	1.339	0.609	2.944	0.467	1.178	0.608	2.284	0.627
Stage (I vs. II)	1.596	0.655	3.890	0.304	1.691	0.683	4.188	0.256
Lymph nodes involved (No vs. Yes)					1.084	0.549	2.141	0.817
Neural invasion	1.232	0.620	2.449	0.551	1.229	0.761	1.985	0.398
PIWIL4 (high vs. low)	2.036	1.025	4.044	0.042	2.185	1.313	3.636	0.003

PFS: progression-free survival; OS: Overall survival; HR: hazard ratio; CI: confidence interval; vs.: versus; cm: centimeters.

**Table 4:** Statistical association between PIWIL3 and PIWIL4 protein expression with clinico-pathological characteristics.

	PIWIL3 Low	PIWIL3 High		PIWIL4 Low	PIWIL4 High	
Parameters	*N* (%)	*N* (%)	*p*-Value	*N* (%)	*N* (%)	*p*-Value
Gender			0.946			0.050
Male	43 (34%)	20 (16%)		34 (26%)	29 (23%)	
Female	44 (34%)	21 (16%)		46 (36%)	19 (15%)	
Age			0.630			0.227
<65 years	18 (14%)	7 (5%)		13 (10%)	12 (9%)	
>65 years	69 (54%)	34 (27%)		67 (53%)	36 (28%)	
Diabetes Mellitus			0.724			0.939
No	59 (49%)	29 (24%)		54 (45%)	34 (28%)	
Yes	21 (17%)	12 (10%)		20 (16%)	13 (11%)	
Stage			0.791			0.204
I	30 (25%)	16 (13%)		26 (22%)	20 (16%)	
II	50 (42%)	24 (20%)		49 (41%)	25 (21%)	
pT			0.503			0.020
I/II	48 (38%)	26 (21%)		40 (32%)	34 (27%)	
III	36 (29%)	15 (12%)		38 (31%)	13 (10%)	
Adjuvant treatment			0.704			0.085
No	50 (51%)	25 (25%)		52 (53%)	23 (23%)	
Yes	17 (17%)	7 (7%)		12 (12%)	12 (12%)	
Size			0.264			0.705
<2 cm	19 (19%)	12 (12%)		19 (19%)	12 (12%)	
>2 cm	50 (50%)	19 (19%)		45 (45%)	24 (24%)	
Lymph nodes involved			0.956			0.713
No	47 (39%)	23 (19%)		43 (36%)	27 (22%)	
Yes	34 (28%)	17 (14%)		33 (27%)	18 (15%)	
Vascular Invasion			0.950			0.875
No	51 (43%)	24 (20%)		46 (34%)	29 (30%)	
Yes	29 (25%)	14 (12%)		27 (25%)	16 (11%)	
Neural Invasion			0.050			0.019
No	27 (23%)	20 (17%)		23 (20%)	24 (20%)	
Yes	53 (45%)	18 (15%)		50 (42%)	21 (18%)	
Grade			0.095			0.917
Low	68 (55%)	37 (30%)		65 (52%)	40 (32%)	
High	16 (13%)	3 (2%)		12 (10%)	7 (6%)	

N: Number of patients; cm: centimeters.

## Supplementary Materials

The following are available online at https://www.mdpi.com/2077-0383/9/5/1252/s1, Supplementary file: Materials and Methods.

## References

[1] Rahib L., Smith B.D., Aizenberg R., Rosenzweig A.B., Fleshman J.M., Matrisian L.M. (2014). Projecting cancer incidence and deaths to 2030: The unexpected burden of thyroid, liver, and pancreas cancers in the United States. Cancer Res..

[2] Tamm E.P., Bhosale P.R., Vikram R., de Almeida Marcal L.P., Balachandran A. (2013). Imaging of pancreatic ductal adenocarcinoma: State of the art. World J. Radiol..

[3] Kelsen D.P., Portenoy R., Thaler H., Tao Y., Brennan M. (1997). Pain as a predictor of outcome in patients with operable pancreatic carcinoma. Surgery.

[4] Oettle H., Post S., Neuhaus P., Gellert K., Langrehr J., Ridwelski K., Schramm H., Fahlke J., Zuelke C., Riess H. (2007). Adjuvant chemotherapy with gemcitabine vs observation in patients undergoing curative-intent resection of pancreatic cancer: A randomized controlled trial. JAMA.

[5] Neoptolemos J.P., Stocken D.D., Bassi C., Ghaneh P., Cunningham D., Goldstein D., Valle J.W., Palmer D.H., Mckay C.J., Doi R. (2010). Adjuvant chemotherapy with fluorouracil plus folinic acid vs gemcitabine following pancreatic cancer resection: A randomized controlled trial. JAMA.

[6] Vera R., Dotor E., Feliu J., González E., Laquente B., Macarulla T., Maurel J. (2016). SEOM Clinical Guideline for the treatment of pancreatic cancer (2016). Clin. Trans. Oncol..

[7] Zeng S., Pöttler M., Lan B., Grützmann R., Pilarsky C., Yang H. (2019). Chemoresistance in Pancreatic Cancer. Int. J. Mol. Sci..

[8] Vagin V.V., Sigova A., Li C., Seitz H., Gvozdev V., Zamore P.D. (2006). A distinct small RNA pathway silences selfish genetic elements in the germline. Science.

[9] Sasaki T., Shiohama A., Minoshima S., Shimizu N. (2003). Identification of eight members of the Argonaute family in the human genome. Genomics.

[10] Farazi T.A., Juranek S.A., Tuschl T. (2008). The growing catalog of small RNAs and their association with distinct Argonaute/Piwi family members. Development.

[11] Gomes Fernandes M., He N., Wang F., Van Iperen L., Eguizabal C., Matorras R., Roelen B.A.J. (2018). Human-specific subcellular compartmentalization of P-element induced wimpy testis-like (PIWIL) granules during germ cell development and spermatogenesis. Hum. Reprod..

[12] Siomi M.C., Sato K., Pezic D., Aravin A.A. (2011). PIWI-interacting small RNAs: The vanguard of genome defence. Nat. Rev. Mol. Cell Biol..

[13] Han Y.-N., Li Y., Xia S.-Q., Zhang Y.-Y., Zheng J.-H., Li W. (2017). PIWI Proteins and PIWI-Interacting RNA: Emerging Roles in Cancer. Cell Physiol. Biochem..

[14] Xie Y., Yang Y., Ji D., Zhang D., Yao X., Zhang X. (2015). Hiwi downregulation, mediated by shRNA, reduces the proliferation and migration of human hepatocellular carcinoma cells. Mol. Med. Rep..

[15] Siddiqi S., Terry M., Matushansky I. (2012). Hiwi Mediated Tumorigenesis Is Associated with DNA Hypermethylation. PLoS ONE.

[16] Gao C., Sun R., Li D., Gong F. (2018). PIWI-like protein 1 upregulation promotes gastric cancer invasion and metastasis. Onco Targets Ther..

[17] Xie K., Zhang K., Kong J., Wang C., Gu Y., Liang C., Qin N., Liu M., Ma H., Dai J. (2017). Cancer-testis gene PIWIL1 promotes cell proliferation, migration, and invasion in lung adenocarcinoma. Cancer Med..

[18] Wang Y., Liu J., Wu G., Yang F. (2016). Manipulations in HIWI level exerts influence on the proliferation of human non-small cell lung cancer cells. Exp. Ther. Med..

[19] Wang Y., Jiang Y., Ma N., Sang B., Hu X., Cong X. (2015). Overexpression of Hiwi Inhibits the Growth and Migration of Chronic Myeloid Leukemia Cells. Cell Biochem. Biophys..

[20] Li W., Martinez-Useros J., Garcia-Carbonero N., Fernandez-Aceñero M.J., Ortega-Medina L., Garcia-Botella S. (2019). The Prognosis Value of PIWIL1 and PIWIL2 Expression in Pancreatic Cancer. J. Clin. Med..

[21] Chen C., Liu J., Xu G. (2013). Overexpression of PIWI proteins in human stage III epithelial ovarian cancer with lymph node metastasis. Cancer Biomark..

[22] Krishnan P., Ghosh S., Graham K., Mackey J.R., Kovalchuk O., Damaraju S. (2016). Piwi-interacting RNAs and PIWI genes as novel prognostic markers for breast cancer. Oncotarget.

[23] Gambichler T., Kohsik C., Höh A.-K., Lang K., Käfferlein H.U., Brüning T. (2017). Expression of PIWIL3 in primary and metastatic melanoma. J. Cancer Res. Clin. Oncol..

[24] Jiang L., Wang W.-J., Li Z.-W., Wang X.-Z. (2017). Downregulation of Piwil3 suppresses cell proliferation, migration and invasion in gastric cancer. Cancer Biomark..

[25] Liu X., Zheng J., Xue Y., Yu H., Gong W., Wang P., Li Z. (2018). PIWIL3/OIP5-AS1/miR-367-3p/CEBPA feedback loop regulates the biological behavior of glioma cells. Theranostics.

[26] Sugimoto K., Kage H., Aki N., Sano A., Kitagawa H., Nagase T. (2007). The induction of H3K9 methylation by PIWIL4 at the p16Ink4a locus. Biochem. Biophys. Res. Commun..

[27] Coley W., Van Duyne R., Carpio L., Guendel I., Kehn-Hall K., Chevalier S. (2010). Absence of DICER in monocytes and its regulation by HIV-1. J. Biol. Chem..

[28] Henaoui I.S., Jacovetti C., Guerra Mollet I., Guay C., Sobel J., Eliasson L. (2017). PIWI-interacting RNAs as novel regulators of pancreatic beta cell function. Diabetologia.

[29] Li L., Yu C., Gao H., Li Y. (2010). Argonaute proteins: Potential biomarkers for human colon cancer. BMC Cancer.

[30] Su C., Ren Z.-J., Wang F., Liu M., Li X., Tang H. (2012). PIWIL4 regulates cervical cancer cell line growth and is involved in down-regulating the expression of p14ARF and p53. FEBS Lett..

[31] Wang Y., Liu Y., Shen X., Zhang X., Chen X., Yang C., Chen X.M. (2012). The PIWI protein acts as a predictive marker for human gastric cancer. Int. J. Clin. Exp. Pathol..

[32] Wang Q., Hao J., Pu J., Zhao L., Lü Z., Hu J. (2011). Icariin induces apoptosis in mouse MLTC-10 Leydig tumor cells through activation of the mitochondrial pathway and down-regulation of the expression of piwil4. Int. J. Oncol..

[33] Heng Z.S.L., Lee J.Y., Subhramanyam C.S., Wang C., Thanga L.Z., Hu Q. (2018). The role of 17β-estradiol-induced upregulation of Piwi-like 4 in modulating gene expression and motility in breast cancer cells. Oncol. Rep..

[34] Wang Z., Liu N., Shi S., Liu S., Lin H. (2016). The Role of PIWIL4, an Argonaute Family Protein, in Breast Cancer. J. Biol. Chem..

[35] Zeng G., Zhang D., Liu X., Kang Q., Fu Y., Tang B., Guo W., Zhang Y., Wei G., He D. (2017). Co-expression of Piwil2/Piwil4 in nucleus indicates poor prognosis of hepatocellular carcinoma. Oncotarget.

[36] Kitagawa N., Ojima H., Shirakihara T., Shimizu H., Kokubu A., Urushidate T. (2013). Downregulation of the microRNA biogenesis components and its association with poor prognosis in hepatocellular carcinoma. Cancer Sci..

[37] Greither T., Koser F., Kappler M., Bache M., Lautenschläger C., Göbel S. (2012). Expression of human Piwi-like genes is associated with prognosis for soft tissue sarcoma patients. BMC Cancer.

[38] Navarro A., Tejero R., Viñolas N., Cordeiro A., Marrades R.M., Fuster D., Caritg O., Molins L. (2015). The significance of PIWI family expression in human lung embryogenesis and non-small cell lung cancer. Oncotarget.

[39] Iliev R., Stanik M., Fedorko M., Poprach A., Vychytilova-Faltejskova P., Slaba K. (2016). Decreased expression levels of PIWIL1, PIWIL2, and PIWIL4 are associated with worse survival in renal cell carcinoma patients. Onco Targets Ther..

[40] Ferreira H.J., Heyn H., Garcia del Muro X., Vidal A., Larriba S., Muñoz C. (2014). Epigenetic loss of the PIWI/piRNA machinery in human testicular tumorigenesis. Epigenetics.

[41] Chen H.-C. (2005). Boyden Chamber Assay. Methods Mol. Biol..

[42] Awasthi N., Zhang C., Schwarz A.M., Hinz S., Wang C., Williams N.S., Schwarz M.N., Schwarz R.E. (2013). Comparative benefits of Nab-paclitaxel over gemcitabine or polysorbate-based docetaxel in experimental pancreatic cancer. Carcinogenesis.

[43] Spratlin J., Sangha R., Glubrecht D., Dabbagh L., Young J.D., Dumontet C. (2004). The absence of human equilibrative nucleoside transporter 1 is associated with reduced survival in patients with gemcitabine-treated pancreas adenocarcinoma. Clin. Cancer Res..

[44] Gao J., Aksoy B.A., Dogrusoz U., Dresdner G., Gross B., Sumer S.O., Sinha R. (2013). Integrative analysis of complex cancer genomics and clinical profiles using the cBioPortal. Sci. Signal.

[45] Cerami E., Gao J., Dogrusoz U., Gross B.E., Sumer S.O., Aksoy B.A. (2012). The cBio cancer genomics portal: An open platform for exploring multidimensional cancer genomics data. Cancer Discov..

[46] Sun Q., Xu W., Ji S., Qin Y., Liu W., Hu Q., Zhang Z., Liu M., Yu X., Xu X. (2019). Role of hepatocyte nuclear factor 4 alpha in cell proliferation and gemcitabine resistance in pancreatic adenocarcinoma. Cancer Cell Int..

[47] Martinez-Useros J., Garcia-Foncillas J. (2016). Can Molecular Biomarkers Change the Paradigm of Pancreatic Cancer Prognosis?. Biomed. Res. Int..

[48] Human hg38 chr12:130329199-130381325 UCSC Genome Browser v396. https://genome.ucsc.edu/cgi-bin/hgTracks?db=hg38&lastVirtModeType=default&lastVirtModeExtraState=&virtModeType=default&virtMode=0&nonVirtPosition=&position=chr12%3A130329199%2D130381325&hgsid=823730117_z8PjbiTzqWd3ZdNvqbZjD0RPpY1I.

[49] Human hg38 chr8:22275316-22357568 UCSC Genome Browser v395. https://genome.ucsc.edu/cgi-bin/hgTracks?db=hg38&lastVirtModeType=default&lastVirtModeExtraState=&virtModeType=default&virtMode=0&nonVirtPosition=&position=chr8%3A22275316%2D22357568&hgsid=813260055_s97A3RqtBxTmk9YMuex2A8Ay0wgb.

[50] Sohn E.J., Jo Y.R., Park H.T. (2019). Downregulation MIWI-piRNA regulates the migration of Schwann cells in peripheral nerve injury. Biochem. Biophys. Res. Commun..

[51] Heyn H., Ferreira H.J., Bassas L., Bonache S., Sayols S., Sandoval J. (2012). Epigenetic Disruption of the PIWI Pathway in Human Spermatogenic Disorders. PLoS ONE.

[52] Abell N.S., Mercado M., Cañeque T., Rodriguez R., Xhemalce B. (2017). Click Quantitative Mass Spectrometry Identifies PIWIL3 as a Mechanistic Target of RNA Interference Activator Enoxacin in Cancer Cells. J. Am. Chem. Soc..

[53] Yang Y., Zhang X., Song D., Wei J. (2015). Piwil2 modulates the invasion and metastasis of prostate cancer by regulating the expression of matrix metalloproteinase-9 and epithelial-mesenchymal transitions. Oncol. Lett..

[54] Ling W., Zhigang H., Tian H., Bin Z., Xiaolin X., Hongxiu Z. (2015). HPV 16 infection up-regulates Piwil2, which affects cell proliferation and invasion in cervical cancer by regulating MMP-9 via the MAPK pathway. Eur. J. Gynaecol. Oncol..

[55] Tan L., Mai D., Zhang B., Jiang X., Zhang J., Bai R., Zhao Q., Li X.X., Yang J., Li D.X. (2019). PIWI-interacting RNA-36712 restrains breast cancer progression and chemoresistance by interaction with SEPW1 pseudogene SEPW1P RNA. Mol. Cancer.

[56] Cheng J., Deng H., Xiao B., Zhou H., Zhou F., Shen Z. (2012). piR-823, a novel non-coding small RNA, demonstrates in vitro and in vivo tumor suppressive activity in human gastric cancer cells. Cancer Lett..

[57] Yin J., Jiang X., Qi W., Ji C., Xie X., Zhang D. (2017). piR-823 contributes to colorectal tumorigenesis by enhancing the transcriptional activity of HSF1. Cancer Sci..

[58] Li D., Luo Y., Gao Y., Yang Y., Wang Y., Xu Y., Tan S., Zhang Y., Duan J., Yang Y. (2016). piR-651 promotes tumor formation in non-small cell lung carcinoma through the upregulation of cyclin D1 and CDK4. Int. J. Mol. Med..

[59] Kalluri R., Neilson E.G. (2003). Epithelial-mesenchymal transition and its implications for fibrosis. J. Clin. Investig..

[60] López-Novoa J.M., Nieto M.A. (2009). Inflammation and EMT: An alliance towards organ fibrosis and cancer progression. EMBO Mol. Med..

[61] Joglekar M.V., Hardikar A. (2010). Epithelial-to-mesenchymal transition in pancreatic islet β cells. Cell Cycle.

[62] Klattenhoff C., Theurkauf W. (2008). Biogenesis and germline functions of piRNAs. Development.

[63] Rojas-Ríos P., Simonelig M. (2018). piRNAs and PIWI proteins: Regulators of gene expression in development and stem cells. Development.

[64] Lee J.H., Jung C., Javadian-Elyaderani P., Schweyer S., Schütte D., Shoukier M., Navernia K. (2010). Pathways of Proliferation and Antiapoptosis Driven in Breast Cancer Stem Cells by Stem Cell Protein Piwil2. Cancer Res..

[65] Rinkevich Y., Voskoboynik A., Rosner A., Rabinowitz C., Paz G., Oren M. (2013). Repeated, long-term cycling of putative stem cells between niches in a basal chordate. Dev. Cell.

[66] Liu W., Gao Q., Chen K., Xue X., Li M., Chen Q., Zhu G., Gao Y. (2014). Hiwi facilitates chemoresistance as a cancer stem cell marker in cervical cancer. Oncol. Rep..

[67] Wang Y., Gable T., Ma M.Z., Clark D., Zhao J., Zhang Y. (2017). A piRNA-like Small RNA Induces Chemoresistance to Cisplatin-Based Therapy by Inhibiting Apoptosis in Lung Squamous Cell Carcinoma. Mol. Ther. Nucleic Acids.

[68] Wang H., Mao C., Li N., Sun L., Zheng Y., Xu N. (2019). A case report of a dramatic response to olaparib in a patient with metastatic pancreatic cancer harboring a germline BRCA2 mutation. Medicine.

[69] Wang H., Word B.R., Lyn-Cook B.D. (2011). Enhanced Efficacy of Gemcitabine by Indole-3-carbinol in Pancreatic Cell Lines: The Role of Human Equilibrative Nucleoside Transporter 1. Anticancer. Res..

[70] Stoffel M., Duncan S.A. (1997). The maturity-onset diabetes of the young (MODY1) transcription factor HNF4alpha regulates expression of genes required for glucose transport and metabolism. Proc. Natl. Acad. Sci. USA.

[71] Hamdan F.H., Zihlif M.A. (2014). Gene expression alterations in chronic hypoxic MCF7 breast cancer cell line. Genomics.

[72] Kiselyuk A., Lee S.-H., Farber-Katz S., Zhang M., Athavankar S., Cohen T. (2012). HNF4α antagonists discovered by a high-throughput screen for modulators of the human insulin promoter. Chem. Biol..

[73] Tissue Expression of PIWIL3—Staining in Pancreas—The Human Protein Atlas version 19.3. https://www.proteinatlas.org/ENSG00000184571-PIWIL3/tissue/pancreas.

[74] Tissue Expression of PIWIL4—Staining in Pancreas—The Human Protein Atlas version 19.3. https://www.proteinatlas.org/ENSG00000134627-PIWIL4/tissue/pancreas.

[75] Egawa S., Takeda K., Fukuyama S., Motoi F., Sunamura M., Matsuno S. (2004). Clinicopathological Aspects of Small Pancreatic Cancer. Pancreas.

[76] Ariyama J., Suyama M., Satoh K., Sai J. (1998). Imaging of Small Pancreatic Ductal Adenocarcinoma. Pancreas.

