# Supplementary material for: The Clinical Significance of PIWIL3 and PIWIL4 Expression in Pancreatic Cancer
Source: J Clin Med. 2020 Apr 26;9(5):1252. doi: 10.3390/jcm9051252 (PMC7287605; doi:10.3390/jcm9051252)
Supplement: Supplementary file 1 [file jcm-09-01252-s001.pdf]

Supplementary Materials: Materials and Methods

Cell Lines and Cell Culture

The human PC-derived cell lines PANC 04.03 (CRL-2555), PL45 (CRL-2558), BxPC-3 (CRL-1687) and one non-tumor human pancreatic ductal epithelial cell line hTERT-HPNE (CRL-4023) were purchased and cultured under American Type Culture Collection (ATCC) recommendations. RWP1 and PANC-1 were kindly provided by Dr. Fatima Gebauer (CRG, Barcelona, Spain). RWP1, PANC-1 cells were routinely grown in RPMI supplemented with 10% fetal bovine serum (FBS) and 1% Penicillin-Streptomycin (P/S). All cell lines were maintained at 37 °C in a humidified atmosphere with 5% CO2.

Patient Samples

A total of 44 pancreatic cancer patients from Hospital Fundacion Jimenez Diaz and 182 pancreatic cancer patients who underwent surgery from 2006 to 2012 were assessed for eligibility. Patients were followed-up until March 2019. Tumors were surgically resected and formalin-fixed and paraffin-embedded (FFPE) immediately for pathologic diagnosis. Tissue microarrays (TMA) were constructed with available FFPE tumor samples. All patients that presented positive margins of resection (R1) were excluded from the study. To assess survival analysis, only patients with available data of progression-free or overall survival were included in the study. Two experienced pathologists reviewed tumor histology (M.J.F.-A. and L.O.-M.).

Ethics Statement

All human samples were kindly supplied by the Biobank of Fundacion Jimenez Diaz Hospital (PT13/0010/0012) and by the BioBank of University Hospital Clinico San Carlos (B.0000725; PT17/0015/0040; ISCIII-FEDER). The institutional review board (IRB) of the University Hospital Clinico San Carlos evaluated the present study, granting approval on Mars 10th, 2017 with approval number nº 17/091-E. The institutional review board (IRB) of University Hospital Fundacion Jimenez Diaz approved the study 15 November 2016 under the approval number 19/16. All patients gave written informed consent for the use of their biological samples for research purposes. Moreover, fundamental ethical principles promoted by Spain (LOPD 15/1999) and the European Union Fundamental Rights of the EU (2000/C364/01) were followed. In addition, all patient's data were processed according to the Declaration of Helsinki (last revision 2013) and Spanish National Biomedical Research Law (14/2007, of 3 July).

Western Blot

Total protein from PC-derived cell lines and controls were extracted with RIPA buffer supplemented with protease inhibitor cocktail (Roche). Samples were fractionated by SDS–polyacrylamide gel electrophoresis and transferred to nitrocellulose membranes (BioRad). Membranes were incubated overnight at 4 °C with the following primary antibodies: PIWIL1(1:500; ab12337; Abcam), PIWIL2 (1:1000; ab181340; Abcam), PIWIL3 (1:100; sc-398779; Santa Cruz Biotechnology), PIWIL4 (1:500; ab111714; Abcam) and Actin (1:10000; a1978; Sigma-Aldrich). To quantify the expression of EMT markers after PIWIL3 or PIWIL4 downregulation, membranes were incubated with the following primary: Fibronectin (1:1000; ab2413; Abcam), Vimentin (1:1000; 5741s; Cell Signaling), E-Cadherin (1:1000; 3195s; Cell Signaling), Occludin (1:1000; sab4200593; Sigma-Aldrich) and Slug (1:1000; ab27568; Abcam). We used an anti-rabbit (NA934V; GE Healthcare) as a secondary antibody for PIWIL1, PIWIL2, PIWIL4, Fibronectin, Vimentin, E-Cadherin, Occludin and Slug, and an anti-mouse secondary antibody (NA931V; GE Healthcare) for PIWIL3 and Actin—both secondary antibodies were conjugated with horseradish peroxidase. For band densitometry we used

the software ImageJ version 1.50i (National Institutes of Health, USA). Protein extracted from mouse testis was used as controls.

RNA Interference

For PIWIL3 and/or PIWIL4 knockdown, we used two independent silencing sequences individually (Thermo Fisher Scientific). All cell lines were firstly transfected at 60%–70% confluence using X-tremeGENE Transfection Reagent (Roche) according to manufacturer's instructions, and a second transfection was carried out in tumor cell lines after 48 h to maximize downregulation. Since PL45 expressed the highest levels of PIWIL3, we have to use a combination of two inhibitory sequences against PIWIL3. Table S1 shows each individual or combination of inhibitory sequences for each cell line according to PIWIL3, PIWIL4 or PIWIL3 and PIWIL4 downregulation. As a control, each cell line was transfected with a scrambled siRNA (sc-37007, Santa Cruz). All subsequent experimental procedures were evaluated at the same day of maximum protein downregulation.

Table S1. Different inhibitory sequences used individually or in combination to downregulate PIWIL3, PIWIL4 or PIWIL3 and PIWIL4.

Cell Line	PIWIL3 Downregulation	PIWIL4 Downregulation	PIWIL3 and PIWIL4 Downregulation
RWP1	s54203 (si03) or s54205 (si05)	s44572 (si72) or s44573 (si73)	s54203 (si03) + s44572 (si72) or s54205 (si05) + s44573 (si73)
PL45	s54203 (si03) + s54204 (si04) or s54204 (si04) + s54205 (si05)	s44571 (si71) or s44573 (si73)	s54203 (si03) + s54204 (si04) + s44571 (si71) or s54204 (si04) + s54205 (si05) + s44573 (si73)
hTERT-HPNE	s54204 (si04) or s54205 (si05)	s44571 (si71) or s44573 (si73)	s54204 (si04) + s44571 (si71) or s54205 (si05) + s44573 (si73)

Wound healing and Boyden Chamber Migration Assay

Cell motility after PIWIL3 and/or PIWIL4 downregulation was estimated by wound healing assays. Cells were grown as a monolayer, and an artificial homogenous wound was created with a sterile plastic 10 μL micropipette tip. The growth of cells in the wound was measured at 0, 6, 12 and 24 h. Boyden chamber migration assays were performed in cell culture inserts with 8-μm pores in 24-well plates (Corning). Cell lines were seeded at a density of 5 × 10^4 cells per insert in 150 μL growth medium without FBS. The recipient wells received 600 μL growth medium supplemented with 20% FBS. The migration was determined after 24 h. Afterwards, cells were fixed and stained with toluidine blue (Sigma-Aldrich). The non-migrated cells on the upper side of the membrane were removed with a cotton swab. Membranes were cut and fixed in microscope slides, and photographs were taken with a stereo microscope (Leica DMi1). Three independent experiments were done, and all experiments were performed in triplicate wells.

Cytotoxicity Assay

Tumor cell lines were treated for 48h with previously determined IC_{50} of Gemcitabine (RWP1:6 nM, PL45: 358nM), Nab-Paclitaxel (RWP1: 11 μM, PL45: 143 μM). Since hTERT-HPNE presented resistance to Gemcitabine, a concentration of 250 μM was used. hTERT-HPNE were also cultured in IC_{50} of Nab-Paclitaxel (236 μM). To determine doses for the combination of Nab-Paclitaxel plus Gemcitabine, IC_{25} dose of Nab-Paclitaxel was set for each cell line due to its high toxicity; then, different concentrations of Gemcitabine were tested to achieve 50% of cell death according to Awasthi N. et al. (42). Therefore, treatment combination for RWP1: 0.36nM of Gemcitabine + 3 μM of Nab-Paclitaxel; for PL45: 156nM of Gemcitabine + 41 μM of Nab-Paclitaxel; for hTERT-HPNE: 14 μM of Gemcitabine + 90 μM of Nab-Paclitaxel. Drugs were kindly provided from the Pharmacology Department of Fundacion Jimenez Diaz Hospital. Cell viability was determined by absorbance with 3-(4,5-dimethylthiazol-2yl)-5-(3-carboxymethoxyphenyl)-2-(4-sulfophenyl)-2H-tetrazolium (MTS) reduction assay (Promega). We performed three replicates of each experiment in triplicate.

Tumor Sphere Formation and Flow Cytometry

PL45, RWP1 and hTERT-HPNE cell lines were separately seeded into ultra-low attachment 6-well plate with 1.5 mL sphere formation medium (SFM) at a concentration of 5000 or 10,000 cells/well, respectively. The SFM consisted of DMEM/F12 medium (11330-032, Gibco) supplemented with 20 ng/mL Epidermal Growth Factor (EGF, 236-EG-200, R&D Systems), 20 ng/mL basic Fibroblast Growth Factor (bFGF, 233-FB-025, R&D Systems), 2% B27 supplement (17504044, Gibco), 1% N2 supplement (17502048, Gibco) and 1% Penicillin-Streptomycin (P/S). Subsequently, cells were cultured at 37 °C in a 5% CO2 humidified environment to form spheroid structures. Photographs were taken with a stereomicroscope (Leica DMi1). Dedifferentiation was evaluated by cytometry to detect CD24+/CD133+/EPCAM+ cells. For this, tumor spheres were dissociated with trysin-EDTA and incubated in presence of the following antibodies: CD24-APC (17-0242-82; BD Bioscience), CD133-FITC(11-1339-42; BD Bioscience) and EPCAM-PE (12-5791-82; BD Bioscience). Cells were then acquired and analyzed on a flow cytometer (FACS Aria II; Becton Dickinson). Three independent experiments were done and all experiments were performed in triplicate.

Immunohistochemistry

Tissue microarrays with 182 patient samples were constructed for immunohistochemistry analysis and contained 364 cores (2 cores per patient) using the MTA-1 tissue arrayer (Beecher Instruments, Sun Prairie, USA). Each core (diameter, 1 mm) was punched from pre-selected tumor regions in paraffin-embedded tissues. Staining was conducted in 2-μm sections. Slides were deparaffinised by incubation at 60 °C for 10 min and incubated with PT-Link (Dako, Denmark) for 20 min at 95 °C in a low pH buffered solution. To block endogenous peroxidase, holders were incubated with peroxidase blocking reagent (Dako, Denmark). Biopsies were incubated for 20 min with a 1:100 dilution of anti-PIWIL1 antibody (ab12337; Abcam), 1:250 dilution of anti-PIWIL2 antibody (ab181340; Abcam), 1:100 dilution of anti-PIWIL3 antibody (ab77088; Abcam), 1:25 dilution of anti-PIWIL4 antibody (ab111714; Abcam) or 1:100 dilution of anti-HNF4A antibody (ab92378; Abcam Cambridge, UK). Tissues were incubated with the appropriate anti-Ig horseradish peroxidase-conjugated polymer (EnVision, Dako, Denmark) to detect antigen–antibody reaction. All antibodies and anti-Ig horseradish peroxidase-conjugated antibody presented high specificity, and no positiveness resulted from these antibodies individually. To determine the best immunohistochemistry conditions, human testis tissues were used as a positive control for PIWIL1, PIWIL2, PIWIL3 and PIWIL4 antibodies and human colon tissues for HNF4A antibody according to The Human Protein Atlas (http://www.proteinatlas.org). Sections were then visualized with 3,3′-diaminobenzidine as a chromogen for 5 min and counterstained with hematoxylin. Photographs were taken with a stereomicroscope (Leica DMi1). To quantify the PIWIL3 and PIWIL4 immunostaining, a semiquantitative HistoScore (Hscore) was calculated. The Hscore was determined by estimation of the percentage of positively stained cells with low, medium or high intensity of staining, after applying a weighting factor following the formula Hscore = (low %) × 1 + (medium %) × 2 + (high %) × 3, and the results ranged from 0–300. To identify the best cut-off point to separate patients according to PIWIL3 or PIWIL4 protein expression and the risk of progression and death on disease, we performed ROC curves. However, the cut-off point according to ROC curves did not separate patients' survival. Therefore, we stratified patients into tertiles according to their PIWIL3 or PIWIL4 Hscore, and the third tertile was considered high PIWIL3 or PIWIL4 expression. HNF4A immunostaining was categorized as positive or negative since HNF4A exhibited a clear nuclear positiveness. Quantification for each patient biopsy was calculated with the average of both cores by two independent researchers.

Statistical Analysis

In wound healing assays, distances between gaps have been measured and a U Mann–Whitney test evaluated differences in length compared to control scramble at 24 h. For evaluation of Boyden chamber assay, cells from each condition from 10 randomly selected fields (10X objective) were

counted and each condition was compared to control scramble with U Mann–Whitney test. Those tumor spheres higher than 70 μm were counted and their sizes were determined by image processing. Statistical analyses between each downregulation and control scramble were assessed with a U Mann–Whitney test. In the cytotoxicity assay, absorbencies were normalized with the absorbance of untreated cells and IC_{50} for each cell line, and treatment was assessed by curve-fitting through nonlinear regression using the Solver tool of Microsoft Excel software. We calculated the drug effect by subtracting absorbance of untreated cells from that of treated conditions. We analyzed differences between each condition and control scramble with non-parametric U Mann-Whitney test. Statistical correlation between hnf4a and hent1 with piwil3 or piwil4 at mRNA level was assessed with Pearson since all variables were normally distributed. Linear correlation was evaluated and interpreted by Pearson's r. Association between HNF4A and PIWIL4 at the protein level was analyzed with Chi-square test. Progression-free survival (PFS) and overall survival (OS) curves according to PIWIL3 or PIWIL4 at mRNA or protein level were performed with Kaplan-Meier, and survival was analyzed with log-rank test. *p*-values ≤ 0.05 were considered statistically significant. All statistics were performed with the IBM SPSS statistics 20.0.
